# Generative AI an academic equalizer? The differential impact of AI-assisted learning on self-efficacy and intrinsic motivation among university students

**DOI:** 10.3389/fpsyg.2026.1779741

**Published:** 2026-03-30

**Authors:** Hongmeng Shao, Rifeng Wang

**Affiliations:** 1 College of Advanced Manufacturing Engineering, Guangxi Science and Technology Normal University, Laibin, China; 2 School of Artificial Intelligence, Guangxi Science and Technology Normal University, Laibin, China

**Keywords:** academic achievement level, educational equity, generative AI, intrinsic motivation, scientific writing, self-efficacy

## Abstract

Generative AI tools have become more common in universities, but studies on the psychology of such use for all students remain insufficiently researched at this time point. This study investigated whether AI-assisted Learning (AIL) is associated with college Students’ intrinsic motivation and Self-efficacy, and if these relationships vary based on Academic performance. A 2 × 2 quasi-experimental design was employed with 200 STEM undergraduates, who were classified into high- and low-achievement groups and assigned to either a DeepSeek-assisted writing condition or a traditional-support writing condition. Intrinsic Motivation and Self-efficacy of Participants after completing the Scientific Paper. The data were analysed by CFA, ANOVA/ANCOVA, mediation analysis and moderated-mediation analysis. The results indicated that the post-tasks’ intrinsic motivation and self-efficacy of students under an AI-accompanied tutoring were much higher than those without it. Further analysis revealed a more pronounced impact of the Group Differences on lower achievers compared to higher ones. Also, intrinsic motivation partly explained why a Writing Support Type was associated with self-efficacy. According to the results, generative AI has equitising characteristics in higher education and provides more robust emotional assistance for weaker learners of initial knowledge.

## 1 Introduction

Recently, in terms of high-end educational applications for genai systems derived from large language model platforms like ChatGPT and DeepSeek has gradually begun to appear. More than half of primary teachers reported that they would like to use these tools; among the 315 participants who answered whether or not, all said yes. Therefore, the use of generative AI has shifted from being auxiliary to help students do their work more effectively to merging with them into learners; that is, its impact on learning becomes increasingly profound at this stage.

One of the significant educational objectives of generating artificial intelligence is its ability for personalisation, adaptability and immediacy of support. Ai-accompanying training immediately gives a response to student doubts, makes one-to-one explanations according to the situation after completing tasks individually. Traditional teaching cannot meet these requirements currently. Functions that can improve the management of complex study tasks or reduce uncertain situations during study. In addition, some students may experience specific psychological issues during the learning process due to these factors; however, it is difficult to determine precisely what kind of problem they are facing based solely on this observation.

Psychological factors that are most directly related to these two aspects among all, self-efficacy and intrinsic motivation. Self-efficacy is learner’s belief in their capability to perform a specific task (Bandura, 1997). It affects efforts, perseverance, strategies for problem-solving and reactions to difficulties. Intrinsic motivation, on the other hand, is driven by a person’s interest, pleasure or satisfaction with learning itself. The two will affect the learning results and self-discipline; harder tasks, such as writing compositions, typically demand them. Those students who are intrinsically driven tend to immerse themselves in their learning more fully. While the ones whose sense of efficacy is high continue until they reach the goal or feel accomplished.

According to the features of generative AI, it is likely positively related to both dimensions. Through personalised feedback, low-stress interactive mode and prompt solutions to problems; the AI-assisted learning will make learners feel competent, self-directed and in charge of the tasks. Experiences that have been acquired may provide theoretical basis for self-determination theory and self-efficacy theory on the promotion of motivation and confidence in support groups. In complex writing tasks, for example, students often struggle with idea organisation, structure, disciplinary expression, and source integration. Utilising the intuitiveness of generative artificial Intelligence (AI) can help overcome certain deficiencies; also able to offer quick answers and support solving problems promptly via AI.

Some existing research has started to describe the educational Value of generative AI in terms of writing assistance, feedback quality, learning engagement and perceived performance. Some researches have shown that AI-assisted Learning may impact the students’ motivation and self-efficacy to some extent. However, at present, there are still some deficiencies in the existing research. Primarily, many research works in this area focus primarily on changes to students’ academic achievements; they rarely look into underlying motivations for using information systems and attitudes regarding system use. Many of the previous researches are based on surveys and descriptions; thus, there is no evaluation about whether AI-assisted learning works in structured tasks. Thirdly, there is no evidence in existing studies that these psychological connections vary among students of varying academic abilities or previous performance.

A major issue immediately before us. While generating intelligent assistance benefits generally; there might be an unequal distribution of this benefit among students. Lower-achieving students are more difficult to motivate sustainably, manage their own motivation better, or complete cognitive-demanding activities on time. Since generative AI offers instant, personalisation and lack of criticism, it will be more beneficial to students in need of reinforcement. The students who have achieved more before may already have better learning strategies, greater self-confidence. This will reduce their relative psychological benefits compared to AI assistance. If this is the case, generative AI has an equalising effect rather than just benefitting one group. It can become closely linked to positive results for students who are relatively disadvantaged in terms of motivation and self-confidence.

Scientific Writing offers an especially suitable context to investigate them. Students’ writing of an academic introduction involves understanding the discipline’s content, organising the thoughts clearly, summarising sources accurately, and expressing arguments formally. These demands make scientific writing both cognitively demanding and motivating for undergraduate students lacking internal motivation. In terms of impact on the creator, generative AI will probably be relatively unimportant in practice as much more time and effort is required for evaluation at present. At the same time, in this Context, it will be examined whether artificial-intelligence-assisted writing is related not only to completing tasks but also to learners’ psychological reactions towards an academically challenging activity.

Against this background, the present study investigates whether DeepSeek-assisted scientific writing is associated with college students’ intrinsic motivation and scientific writing self-efficacy. Furthermore, this study investigates whether the associations among these factors differ by academic ability levels; if intrinsic motivation acts as a mediator bridging Writing support Type and Self-efficacy. Therefore, this study has provided contributions to existing research in the following aspects. First, it extends the literature on generative AI in higher education by focusing on psychological outcomes rather than performance alone. Secondly, it establishes a Theory-Informed framework that connects AI-assisted learning with intrinsic motivation and self-efficacy. Thirdly, investigate whether there is an advantage in terms of psychology for the assistance provided by AI in learning for low-performing students. This can help answer whether generative artificial intelligence has a compensation function in education.

Thus, in response to the following research issues:

(1) whether DeepSeek-assisted writing is associated with students’ intrinsic motivation and self-efficacy;
(2) whether these associations vary by academic achievement level;
(3) whether intrinsic motivation mediates the association between writing support type and self-efficacy.

## 2 Literature review

### 2.1 Theoretical foundations

As an educational tool, AI-generated content will be more frequently used by teachers in the classroom to address student questions or tasks. At the same time, there are various factors related to it. Given that this study focuses on students’ intrinsic motivation and scientific writing self-efficacy, the theoretical basis of this paper is largely based on SDT and self-efficacy theory, supplemented by insights from Cognitive Load Theory (CLT), scaffolding learning theories, etc. Together, they can explain how artificial intelligence-supported learning might decrease the felt task difficulty to promote positive psychological reactions under stress in learning tasks.

#### 2.1.1 Self-determination theory

According to Self-Determination Theory, extrinsic motivation tends to arise in the absence of students’ fundamental psychological needs of autonomy, competence and sense of belonging (Deci and Ryan, 2000; Ryan and Deci, 2000; Deci and Ryan, 2013). In education, students are likely to be more engaged and committed if they feel a sense of agency and efficacy in their participation, as well as supportive learning environments providing the necessary help.

Generative AI is likely applicable across all these requirements. AI tools enable learners to explore the materials flexibly, express themselves freely by asking questions of their own initiative, and learn independently. Secondly, adaptive feedback, real-time explanations and personalisation will boost students’ feelings of competence by showing them the results after their own solutions. In addition, though AI cannot replace the genuine assistance from others in daily life and learning for students’ emotional development, it can offer some low-stress forms of communication that make them feel supported temporarily.

SDT-based mechanisms have particular relevance to the uncertainty of structure, expression and idea development in academic Writing that many students face. When learners perceive the task as being within their control and of suitable difficulty for them, they are more likely to participate willingly and continue engaging. Recently, some scholars have proposed that personalised feedback generated by AI can enhance students’ motivation to learn and goal-directed behaviours through autonomy- and competence-enhancing functions. In the related research of Generative AI application in universities, there is motivation. This study also shows that it affects students’ use and gains benefits from such systems.

#### 2.1.2 Self-efficacy theory

Based on self-efficacy theory, individuals’ beliefs about their capabilities in realising the objectives have a significant influence on attitudes, behaviour patterns, learning outcomes, persistence of actions and feelings. Self-efficacy has become an important consideration for whether more difficult problems should be chosen by students and how long they will persist while solving issues in class over the years. Self-efficacy in writing contexts also has a close connection with self-control, strategic behaviour and educational outcomes (Zimmerman and Bandura, 1994; Golombek et al., 2018).

Generative AI may have a higher level of self-efficacy due to an increased opportunity for partial success and reduced uncertainty about the task. When students get instant correction and understanding, they will have more sense of achievement in mastering new knowledge or skills; thus, their self-efficacy is improved. This mechanism is very suitable for writing tasks; learners require assistance with ideas arrangement, academic exposition as well as structure building.

Recently, some other studies also support this conclusion. Writing-Related Contexts: Generative AI has been associated with improved Writing Self-Efficacy via real-time assistance and process-oriented support (Pellas, 2023; Bouzar, 2024; Huang and Mizumoto, 2024). The same phenomena have also appeared in other fields such as design education (Hwang and Wu, 2025); Entrepreneurship education; Chatbot based on academic support (Tanveer et al., 2024) and Programming Education (Yilmaz and Karaoglan Yilmaz, 2023). There is meta-analytical support for the general association of AI-enhanced intervention self-efficacy effects: Positive impacts have been reported.

#### 2.1.3 Cognitive load theory and scaffolded learning

According to cognitive-load theory, there is still an issue of influencing users’ motivation and efficacy with generation-AI. As per the CLT, learning is affected by an overabundance of extraneous loads on students’ cognitive abilities beyond what they can manage appropriately, thus reducing meaningful learning opportunities for them (Sweller, 1988; Paas et al., 2003).

Generative AI can reduce learners’ unnecessary cognitive burden to some extent through assistance such as task breakdowns, organisational structures of information, summarisation of primary contents, and real-time resolution of doubts. These Functions are particularly important for academic Writing Where students encounter challenges at the same time regarding topic Selection Structural arrangement Language use Reference citation. Research on second language academic Writing and other cognitively demanding Learning scenarios indicates that decreasing unessential processing demands may boost task Engagement and learning results (Nawal, 2018; Millin et al., 2020). Recently, some studies have shown that, based on the theory of cognitive load, scaffolding that helps learners manage complex tasks better is more effective.

Also in line with the concept of scaffoldled instruction at large. Students who cannot finish a task by themselves can still get help to bridge their current level of ability with the required skills for completion. Thus, generative AI serves as a support system that offers relevant and timely help in times of distress. When there is an appropriate reduction in cognitional load of complicated operations among students, they can recover some potential due to prolonged engagement with such tasks. Consequently, there will be greater likelihood that both kinds of factors arise in the process.

#### 2.1.4 A comprehensive theoretical perspective

In light of SDT, self-efficacy theory and CLT/scaffolded learning’s contributions to enhancing learners’ psychological well-being in the context of generative AI technology application. AI-assisted learning may enhance the sense of control and accomplishment among students; reduce cognitive strain in learning; more opportunities for experiencing mastery. Through these ways, the generation of intelligent systems can bring more inherent motivation and confidence to students, especially for intellectually challenging work like scientific papers.

### 2.2 Applications of generative AI in higher education

Generative AI is now widely appearing in higher education, mainly showing up in writing, programming, Design, problem-solving and self-directed learning. Based on institutional analysis, university-wide policies in many countries have been established to address the issue of Gen-AI use more scientifically (Jin et al., 2025). Research at the students’ level shows that AI has gradually been recognised by them more than an information-acquisition device as a tool to generate ideas, provide assistance for tasks and enhance learning efficiency.

One of the strongest points that are frequently mentioned about generator AI’s ability to give individual assistance. Unlike the static teaching aids, AI system will respond flexibly to learners’ responses; it can explain in various difficulty levels based on their questions and development process. Personalised service can drive up customer traffic and improve participants’ sense of purpose. Mohammed reported enhanced tasks-driven abilities due to the training programme (Mohamed et al., 2024). Systemic Reviews also indicate a positive impact of AI-supported Learning on Students’ outcomes in diverse Areas of Study at the tertiary level (Qian, 2025; Chen and Cheung, 2025).

Secondly, its basic feature is real-time. Classroom traditional teaching is usually late in providing feedback; however, Generative AI can offer it promptly after multiple inquiries. At this time, the value of such works is raised in relation to events that take place unexpectedly. Studies on the support for AI-assisted writing and revision have shown that immediate assistance can help enhance both task organisation and students’ perceptions of their work during tasks (Pellas, 2023; Bouzar, 2024; Huang and Mizumoto, 2024).

A third feature concerns the interactional climate of AI-supported learning. As AI interaction is usually seen as a non-pressure or unjudgmental environment; some students may be more at ease in posing inquiries, testing ideas, etc., and also freely make errors. It would also serve these students well when facing difficulties they cannot explain in person. Research on generative AI in education shows that students often appreciate the flexibility, response speed and perception of safety offered by AI assistance (Pang and Wei, 2025).

At this time, there are also many worries about how generative AI will be used in universities currently. Broader worries about educational equity also imply that the advantages brought by artificial intelligence will be influenced by institutional resources, technological accessibility and execution quality (Gabriel, 2024; Wilmers, 2024).

### 2.3 Generative AI and students’ self-efficacy

There is a growing body of evidence suggesting that generation AIs are linked to students’ sense of efficacy in various subjects. Such a mode tends to appear more frequently in tasks that demand continuous exertion, multiple revisions, and handling affairs; writing, design, programming or entrepreneurship can be seen.

By utilising artificial intelligence, teachers and students will be better able to manage the Study process. Provide immediate encouragement, explain errors clearly and demonstrate appropriate examples in order to help users understand more easily. When students see their own improvement and achievement in some parts, they will gradually feel that they can tackle similar problems later on by themselves. This interpretation aligns with mastery-based accounts of the development of self-efficacy (Bandura, 1997).

During writing times, it holds special application values. Research indicates that generation AI can enhance the writing efficacy of students in their ability to revise language, organise concepts, and arrange structures better (Pellas, 2023; Bouzar, 2024; Huang and Mizumoto, 2024). For example, as reflected in the same trend of AI-assisted peer review and academic papers: More autonomous revision ability and self-regulation capability were developed under automated annotation support. There are also studies showing that AI-enhanced task-based language learning can promote better English-language writing self-efficacy in postgraduate students’ minds (Hao et al., 2026).

Generative AI beyond writing is also related to self-efficacy in other fields. Design Education: AI helps enhance creative cognition in some way by promoting the process of self-efficacy; Hwang and Wu’s research. AI support in entrepreneurship education is associated with more pronounced entrepreneurial intent via self-efficacy and perception of university support (Xie and Wang, 2025). Programming Education: AI-Based tools are associated with higher programming self-efficacy and improved computational thinking skills among participants (Yilmaz and Karaoglan Yilmaz, 2023). AI-based Chatbot has also improved the academic motivation of University Students, as demonstrated by Tanveer et al. (2024).

At this time, the relationship between AI and self-efficacy is unclear at all times. Some scholars have pointed out that there may be a paradox of increased confidence among students to complete tasks but also technology dependency due to over-reliance on AI (Zhang and Xu, 2025). It can be inferred from this that higher self-efficacy is more likely to occur under the condition of strong AI support in learning rather than independence.

Based on the general situation described in various publications; Generative AI may affect students’ autonomous learning to some extent by giving prompt explanations along every challenge during their studies.

### 2.4 Generative AI and intrinsic motivation

Besides self-efficacy, students’ intrinsic motivation may also be related to their experience of generative AI. Intrinsic motivation refers to engaging in learning for its own sake due to interest, pleasure or intrinsic satisfaction (Deci and Ryan, 2000; Ryan and Deci, 2000). It serves as the primary motivator for perseverance, mastery-seeking behaviour, and autonomous regulation.

SDT from this standpoint may promote intrinsic motivation through enhancing people’s sense of self-determination and capability-building. Attracting students’ attention through providing more choices, enabling each student to make decisions on their own about the following operations in the system. Also at this time, immediate assistance and adaptability in prompting can help learners feel more capable of success. Based on research, AI-guided personalised feedback and AI-assisted learning environment have made the tasks more manageable and meaningful to enhance students’ motivation in learning (Xu et al., 2025).

Evidence has consistently indicated positive motivational effects recently. Systematic review and Meta-analysis indicate that generative AI may have a positive impact on the motivation and enthusiasm of students studying at higher learning institutions (Xia et al., 2025; Qian, 2025; Chen and Cheung, 2025). Experiments and quasi-experiments also showed that students’ motivation increased when using AI-assisted teaching compared to the conventional method (Tang, 2025; Huang and Mizumoto, 2024). Generative AI in Design and Art Education is closely connected with the outcome of achieving achievements as well as students’ sense of motivation and self-efficacy (Chen J. et al., 2025; Chen R. et al., 2025). Research on the application of AI also shows that learners’ motivation in learning directly affects their use and acceptance of generative AI teaching assistants (Tang, 2025).

Another possible mechanism concerns emotional experience during task completion. Generative AI can provide a low-stress, non-judgmental interactive situation that reduces student anxiety; therefore, it encourages students to explore more, correct errors bravely, and persevere. Other people’s opinions may still have an impact on it due to severe issues. Therefore, students may feel that AI-assisted learning is more accessible and acceptable to them, thereby increasing their intrinsic motivation further.

Moreover, there are also some restrictions in previous research. Although the application of artificial intelligence can increase students’ interest in completing tasks. Students become too dependent to be active learners who develop innovation and creativity independently. This has been described as a possible paradox of improved task management accompanied by reduced independent agency (Zhang and Xu, 2025). Thus, the motivational implications of AI may depend on whether it is used to support student agency or to replace it.

### 2.5 Differential associations across academic achievement levels

While there is still a lack of consistency in how various users benefit from generative AI. A significant new problem that has arisen in the research concerning whether AI assistance is significantly related to better results among certain groups of students, particularly those with varying degrees of initial knowledge.

Low-achieving students frequently encounter difficulties due to various reasons. They may have fewer previous successes, be more uncertain, lack good learning methods, and feel anxious in handling complex tasks. Research on academic self-efficacy has shown that prior competence-related experience is closely related to students’ educational confidence and expectations of their own education (Kramer et al., 2025). At present, when such an urgent task arises, a short-term, specific operation can be scheduled in advance to explain step by step and provide less scary treatment so that people feel more willing to try it.

Empirical studies have shown that AI-assisted Support can be particularly beneficial for some students who require more help. Chen J. et al. (2025) and Chen R. et al. (2025) also discovered that the utilisation of Generative AI can improve student performance, interest and self-efficacy in Design-Arts courses. Research in the broad field of education equity shows that technological assistance can enhance the Teaching and learning environment for underprivileged students when distributed fairly (Chen J. et al., 2025; Chen R. et al., 2025). Even though some parts of this work do not explicitly address scientific writing or the use of generative AI in structured tasks directly; nevertheless, it suggests that with AI-based assistance learners might compensate for disadvantages caused by structural or academic limitations.

On the other hand, outstanding students have developed better learning strategies, enhanced self-confidence in their abilities to learn independently, etc. For such students, AI may still be useful, but the relative psychological advantage may be smaller because they begin from a higher baseline. Also, according to some research findings on whether high-efficiency students might lose their sense of autonomy because of excessive assistance from artificial intelligence, there is a trend of diminishing returns in these cases (Zhang and Xu, 2025; Wilmers, 2024).

At this time, individual differences not related to academic performance can also affect AI-related results. Experiments suggest that learners’ learning-related characteristics affect how they interact with tasks and processes information (Khayr et al., 2023). According to the relevant works, the students’ attitude towards AI and its use may affect learning outcomes indirectly through cognitive mechanisms (Liu et al., 2025) or motivationally based pathways. Based on these results, academic achievement level may serve as a potential, yet not the only, modulating factor for AI-assisted learning effects.

Research on this point can lead to suggestions for its ability to affect equity in higher education through generative AI. Rather than simply benefiting learners on average, AI may be more strongly associated with positive psychological outcomes among students who are initially less advantaged in confidence or academic readiness. But caution is required in interpretation here. Scholars of educational inequality have pointed out that the outcomes of AI use are subject to factors including technical accessibility, institutional guarantees, digital skills promotion, and practical effectiveness (Gabriel, 2024; Wilmers, 2024).

### 2.6 Research limitations and the present study

There are currently many deficiencies in the research on creating artificial intelligence for college students’ learning.

Many of the previous researches’ focus has been primarily on performance outcomes, technology adoption or people’s attitude towards AI; comparatively few research have investigated the fundamental motivational components underlying them, including intrinsic motivations and self-efficacy in an all-encompassing theoretical perspective. The above factors will help retain students’ interest, improve the ability to self-discipline in studying and conquer difficulties more easily.

Secondly, while numerous researches focus on the advantages of personalised teaching and immediate feedback by generation-ai systems, little is known about how these effects operate psychologically in students. Specifically, how much influence intrinsic motivation has on the association among AI-assisted learning, self-efficacy cannot be found in existing studies.

Third, more studies show that there are differences between learners of various types when using AI; however, systematic testing of these effects is lacking in college-level tasks. Specifically, how much better low-achieving students feel psychologically after using AI-supportive learning compared to high-achieving students has received insufficient attention.

Fourthly, Writing has become a common and meaningful domain for educational research with AI generation; however, most existing works are mainly in the form of description and policies that do not directly assess individuals’ emotional changes during this process. It is worthy of note that scientific writings have relatively high cognitive and motivational requirements for students, making them suitable for studying the relationship between AI-assisted learning and intrinsic motivation or self-efficacy among learners.

Based on this, this paper explores whether DeepSeek-assisted scientific writing positively correlates with university students’ motivation for scientific work and self-efficacy in science writing. Additionally, does academic achievement level moderate the relationship between writing support types and self-efficacy; and is intrinsic motivation a mediator that links writing support type to self-efficacy?

### 2.7 Research hypotheses

Based on the theoretical framework and previous research summarized in this paper, some hypotheses are proposed here.

*H1*: Students in the DeepSeek-assisted writing condition have a greater degree of post-tasks internalisation than those under traditional support.

*H2*: The DeepSeek-assisted writing group is expected to have a greater increase in scientific writing self-efficacy than those under regular support.

*H3*: Academic achievement levels are expected to buffer the relationship between the writing-support types and the two psychological outcomes, such that under a DeepSeek-assisted condition, there would be greater post-task benefits for low-achieving students than for high-achieving ones.

*H4*: Intrinsic motivation will mediate the relationship among writing support type, scientific writing self-efficacy.

## 3 Method

### 3.1 Participants

Recruits of the participants were undergraduate students majoring in science, technology, engineering, and mathematics from a scientific writing course at this Chinese university. To reduce the risk of confounding due to substantial prior writing experience, only STEM students were enrolled, and those who had published scientific papers before were excluded. The selection of this sample design aimed to enhance the consistency among subjects, ensuring that all members had more similar characteristics in terms of basic level learning abilities and writing skills.

In all, 200 students were surveyed for this study; there were 112 boys and 88 girls in total. The average age of the participants was 21.2 years (standard deviation = 1.6). In terms of students’ grades, there are 48 s-year students (24.0%), 102 third-year students (51.0%), and 50 fourth-year students (25.0%).

#### 3.1.1 Academic achievement grouping

To investigate whether the association between AI-assisted writing was moderated by academic achievement levels; participants were divided into high and low-achieving groups based on their accumulated weighted GPA. GPA data was sourced from official student records. Following a median-split procedure, students in the upper 50% of the GPA distribution were categorized as high-achieving, whereas those in the lower 50% were categorized as low-achieving. Through this process, two equivalent-ability achievement groups were formed to explore if there is a relationship among the effect of writing support types on psychological changes at different levels of students’ initial learning;

#### 3.1.2 Recruitment and ethical approval

Voluntariness of participation. At the beginning of this study, it was explained in detail to students about its aim, process, and right for withdrawal as long as they wanted. Participants provided written informed consent before the collection of the data.

#### 3.1.3 Instructional context

All participants were taken from the same scientific writing course and therefore shared an instructional setting during this research. Based on this, scientific writing has been defined as a cognitive-physically demanding activity involving ideas organisation, disciplinary reflection, formality expression and the synthesis of diverse sources. Therefore, it offers a suitable environment to investigate the effect on students’ motivation and self-assessment by means of various types of written communication when they finish tasks. Exclude students who have previously published to reduce possible bias due to superior writing ability.

### 3.2 Research design

Using a 2 × 2 quasi-experiment between-subjects design, this study aimed to explore whether post-task intrinsic motivation and scientific writing self-efficacy varied according to the Writing Support Type and Academic Achievement Level after completing a scientific writing task.

The two factors were as follows:

Writing Support Type.
DeepSeek-aided Writing Conditions.
Traditional-support condition.
Academic Achievement Level.
High-achieving students.
Low-achieving students.

The two dependent variables were:

Intrinisic motivation.
Scientific writing self-efficacy.

The Design could explore how the primary influences of Writing Support Type and Academic Achievement Levels were exhibited in both post-task Psychological Outcomes; its interactions among them can also be observed. Additionally, it was determined whether intrinsical motivation mediates the relationship among writing support type, self-efficacy and academic achievement levels; and investigate whether there is an interaction effect of academic ability on these relationships.

Since the study needed subjects under both conditions to interact with an explicitly generated-ai device unblinded is impossible. The participants would have known that they were performing tasks with deepseek assistance or without any other form of help. Therefore, in this sense, it can be considered to be quasi-experiment design rather than blind experimental design.

#### 3.2.1 Independent variable and moderator

1 Support for writing.

The participants under DeepSeek help finished their writing tasks more rapidly and with fewer attempts compared to controls who were not guided by DeepSeek. DeepSeek was used in this paper for ideas generation, organisation of contents, integration of literature, logical development, academic expressions etc., throughout the process of scientific writing.

Participants in the traditional-support condition completed the same writing task using conventional non-AI resources. They could access academic databases and writing guidelines; however, there was no real-time generated-ai response, adaptability recommendations or interactional language services provided by these during the assignment.

Therefore, there was a difference between these two states that did not lie in different tasks. Both groups were given the same writing task under similar course contexts and timing regulations, but one group obtained interactive AI-scaffolded support while the other used only static, unassisted materials.

2 Academic achievement level.

Academic achievement was assessed by cumulative weighted GPAs, divided into high-academic and low-academic levels according to the sample median. The Grouping Variable was added here to explore whether the impact on Students’ Writing Development, depending upon their pre-school reading level;

#### 3.2.2 Dependent variables

1 Intrinsic motivation.

Intrinsic motivation refers to students’ specific interests, pleasures and involvement in scientific writing activities within an objective. It is worth noting that this study refers to it as a post-tasksmotivationalState following the Task-Completed theory.

2 Scientific writing self-efficacy.

Scientific writing self-efficacy referred to students’ confidence in their ability to complete the scientific writing task successfully. At present, most studies focus on organisational Confidence of making presentations. They adopt appropriate academic language and organise associated contents in a systematic way.

#### 3.2.3 Covariates

The following variables that might have caused bias due to confounders were excluded from analysis by controlling for them: Cumulative weighted GPA, Task awareness, Grade Level, gender.

These variables have been statisfically adjusted in the ANCOVA model of intrinsic motivation and scientific writing self-efficacy.

#### 3.2.4 Participant allocation

First, classify the participants into high-and-low achievement groups according to their cumulative weighted GPAs. Then, they were assigned in a fair way across the two writing support conditions to form a 2 × 2 Design of cell size equated cells. Through this way, each group had fifty participants.

Because assignment is embedded in an authentic teaching situation with real operational conditions, the distribution process should not be treated as a rigorous form of complete randomisation experimentally. Instead, a goal was set to increase comparability between the Conditions and maintain balance in the Cells.

The final distribution of the participants is as follows: Table 1.

**Table 1:** Participant distribution across writing support type and academic achievement level.

Academic achievement level	DeepSeek-assisted condition	Traditional-support condition	Total
High-achieving students	50	50	100
Low-achieving students	50	50	100
Total	100	100	200

### 3.3 Procedure

The study was carried out in the computer Laboratory within a standardised learning Environment. About 90 minutes of training per session was arranged in sequence as follows: orientation and task description, work implementation, and after-work evaluation. All participants used similar computer hardware and were in the same environment as those without other contexts for reduction.

#### 3.3.1 Stage 1: orientation and task introduction

At the start of every Session, introduce in general outline to the participants’ task content’s requirements. Participants completed the short questionnaires of demographics: age, gender, grade level and tasks’ familiarisation firstly.

Then, introduce the scientific paper creation assignment to participants. Ask them to write a 500-600-word, IEEE-formatted abstract for a hypothetical study on developing a lightweight image-recognition algorithm with attention mechanism capabilities in their own research project. Task materials included background information, performance comparison against traditional architectures, essential readings and summaries of this research’s unique contributions to existing studies. All the participants were given identical task material and writing instructions.

#### 3.3.2 Stage 2: writing task execution

The participants wrote separately in different conditions as specified by them.

DeepSeek-assisted condition.

Student groups using DeepSeek-assisted conditions engaged with it while completing their writings. Be encouraged to use this system as a reference for clarifying ideas, arranging material contents, editing scholarly language, revising argumentation logic, etc. Reduce the dependence of passively accepting artificial intelligence-generated outputs; have them review, modify in multiple iterations their original evaluations based on these responses and incorporate existing information accordingly.

Traditional-support condition.

Students in the traditional-support condition completed the same writing task without access to generative AI tools. Academic Databases and the Writing- Guide Document provided by the computer science field have been permitted as necessary supporting resources. But there was no real-time adaptive prompt or AI suggestion for the user’s work.

#### 3.3.3 Stage three: post-task assessment and debrief

After finishing the writing task immediately, they answered the questionnaires about intrinsic motivation and scientific writing self-confidence. Collected all the materials and provided some time for them to be permeated; then arrange a brief Summary at that time after concluding this session. To ensure transparency and respect the subjects’ rights to know this step was designed.

### 3.4 Strategies

Ensure that the language used in the survey is appropriate for China after translation by back-translating them again after a pilot test. Each piece of content will receive a rating based on the following scale: strongly disagree is scored as 1 point, whereas totally agree receives seven points.

#### 3.4.1 Intrinsic motivation

Intrinsically motivated was assessed through six items modified from the Interest/Enjoyment sub-scales in IMI, which were adjusted for use in the scientific writing context. Students’ interest and participation in writing; scale assessment to determine this value. Sample item: “I found the process of writing this Introduction to be enjoyable.”

In a pilot study with 30 science-technology-innovation-engineering-art (STEM) students, the scale showed good internal consistency (Cronbach’s alpha = 0.91). Reconfirming the internal consistency reliability and convergent validity of the test based on the entire dataset from this research is presented in the result part.

#### 3.4.2 Scientific writing self-efficacy

The scientific writing self-efficacy scale was constructed by adapting a number of existing academic self-efficacy constructs into the context, with 6 items. Items to evaluate the students’ confidence of constructing logical arguments, proficient use of appropriate academic language, integrating complex materials into an outline logically. A sample item is: “I am confident that I can construct a logical argument.”

The scale showed good test–retest reliability in the pilot study (Cronbach’s *α* = 0.93). Reliability and Validity were also assessed in the main study; all the results are presented in the Results section.

#### 3.4.3 Reliability and construct validity assessment

Used Cronbach’s alpha to assess the level of internal consistency for this study. The investigation of conformity validity used confirmatory factor analysis (CFA) in AMOS 26. CFA specifies that the underlying constructs of these 12 observed indicators are intrinsically motivated (i.e., “intrinsic”) or based on an assessment of science writing skills (“scientific”).

Multiple fit indicators were used to evaluate model adequacy: χ^2^/df; CFI, TLI, IFI, RMSEA, SRMR, GFI, NFI, etc. Additionally, AVE, CR, and composites correlation will be used in assessing the convergence and divergent validity of internal consistency tests here. Information is likely to appear in this Section.

### 3.5 Data analysis

Data were analysed using SPSS 27.0 and AMOS 26. To evaluate the psychometric properties of each component scale in turn, examine changes among groups after task completion; verify whether the proposed mediated-and-modified relationship holds.

#### 3.5.1 Preliminary analyses

First, descriptive statistics were calculated separately by group for intrinsic motivation and science Writing Self-efficacy. Cronbach’s α coefficient to measure internal consistency. In order to verify construct validity through confirmatory factor analysis of the two-dimensional measurement model.

#### 3.5.2 Assumption verification

First, checks for distributions prior to any analysis was done; normality was tested by means of Shapiro–Wilk and Kolmogorov–Smirnov tests, and homogeneity of variance by Levene’s test.

#### 3.5.3 Main and interaction effects

Firstly, to examine whether there are differences in the two dependent variables under different levels of writing support type and student academic achievement levels using MANOVA. Subsequent univariate ANOVA tests were conducted on the variables of intrinsic motivation and scientific writing self-efficacy. Interaction effects have been investigated using simple-effects analyses and interaction plots.

#### 3.5.4 Covariate-controlled analyses

To check if the observed group disparities disappeared following correction for these confounders, analyses of covariance (ANCOVA) were performed on inquiring attitude towards online quizzes and the ability to write scientifically; GPA was used as a covariate.

#### 3.5.5 Mediation analysis

To determine if intrinsic motivation mediates the effect of writing support on scientific Writing self-efficacy; a mediation analysis using Process macro model 4 was conducted. Writing support type was used as the predictor, intrinsic motivation as the mediating variable, and self-efficacy as the outcome variable. Estimate the indirect effect through 5,000 bootstrapped samples and provide a 95% Confidence Interval. Indirect effects were considered statistically significant if they fell outside the 95% confidence interval for non-significance.

#### 3.5.6 Moderation analysis

To determine if academic achievement level mediated the relationship between writing-support type and outcomes, multiple-regression analysis for moderation was used to check this. Writing-support type, academic achievement level, and their interaction term were entered as Predictors. When the interaction effect of writing support type was significant, simple-effect analysis compared the associations among outcomes at different achievement levels.

## 4 Results

### 4.1 Descriptive statistics and measurement quality

#### 4.1.1 Descriptive statistics

Descriptive statistics were calculated for intrinsic motivation and scientific writing self-efficacy in the traditional-support condition and the DeepSeek-assisted condition. According to Table 2, after DeepSeek assistance, students’ post-tasks were more intrinsically motivated and had greater self-efficacy compared with those who received only traditional support.

**Table 2:** Descriptive statistics for intrinsic motivation and scientific writing self-efficacy by writing support type.

Writing support type	Dependent variable	M	SD	Minimum	Maximum	95% CI	Skewness	Kurtosis
Traditional support	Intrinsic_Motivation	4.07	1.13	1.67	6.50	[3.84, 4.29]	0.03	−0.70
DeepSeek-assisted	Intrinsic_Motivation	5.78	0.77	3.67	7.00	[5.63, 5.94]	−0.45	−0.42
Traditional support	Self_Efficacy	3.61	1.05	1.33	5.67	[3.40, 3.82]	0.03	−0.71
DeepSeek-assisted	Self_Efficacy	5.46	0.80	3.67	7.00	[5.30, 5.62]	−0.23	−0.80

For intrinsic motivation, the traditional-support condition had a mean score of 4.07 (SD = 1.13, 95% CI [3.84, 4.29]), whereas the DeepSeek-assisted condition had a mean score of 5.78 (SD = 0.77, 95% CI [5.63, 5.94]). For scientific writing self-efficacy, the traditional-support condition had a mean score of 3.61 (SD = 1.05, 95% CI [3.40, 3.82]), whereas the DeepSeek-assisted condition had a mean score of 5.46 (SD = 0.80, 95% CI [5.30, 5.62]).

#### 4.1.2 Reliability and preliminary validity evidence

Internal consistency assessment based on Cronbach’s alpha for both scales in this study. According to the results, there were extremely high internal consistencies of intrinsic motivation (*α* = 0.958) and scientific writing self-efficacy (0.960). Corrected Item-total correlations were also quite high across all items (0.857–0.880, intrinsic motivation; 0.859–0.882, scientific writing self-efficacy); alpha coefficients did not increase after deleting one of these questions. This indicates excellent reliability, but due to its extremely high alpha coefficient, it suggests that many of these measures might be highly correlated across all items.

Initial factor reliability examination before performing confirmatory factor analysis. The KMO coefficient reached 0.956, and the Bartlett’s test of sphericity had a significant result: χ^2^(66) = 2643.316, *p* < 0.001. The data were applicable to factor analysis. Principal component analysis with varimax rotation explained 83.19 per cent of the total variance in this study. The six self-efficacy items loaded primarily onto one factor, and the six intrinsic motivation items loaded mainly into another; this provides some support that these are empirically different concepts based on the data collected. As shown in Table 3.

**Table 3:** Reliability and initial factorability outcomes.

Indicator	Result
Cronbach’s α for intrinsic motivation	0.958
Corrected item–total correlations for intrinsic motivation	0.857–0.880
Cronbach’s α for scientific writing self - efficacy	0.960
Corrected item–total correlations for scientific writing self - efficacy	0.859–0.882
KMO	0.956
Bartlett’s test of sphericity	χ^2^(66) = 2643.316, *p* < 0.001
Number of extracted components	2
Variance explained by Component 1	68.74%
Variance explained by Component 2	14.44%
Total variance explained	83.19%

### 4.2 Confirmatory factor analysis and construct validity

Confirmatory factor analysis (CFA) was performed to verify the factorial validity of the hypothesised two-factor structure, which included intrinsic motivation and scientific writing self-efficacy.

#### 4.2.1 Measurement model fit

All standardised factor loadings were statistically significant (*p* < 0.001) and ranged from 0.879 to 0.904, indicating that all items loaded strongly on their intended latent constructs. The overall model fit index also showed a good match with the data: χ^2^/df = 1.117; RMSEA = 0.024; SRMR = 0.022; CFI = 0.998; TLI = 0.997; IFI = 0.998; GFI = 0.954; and NFI = 0.978. Together, they provide support for the adequacy of the proposed two-factor measurement model. As shown in Table 4.

**Table 4:** Model-fitting indicators of the two-factor measurement model.

Metric	χ^2^/df	RMSEA	SRMR	CFI	TLI	IFI	GFI	NFI
Measurement value	1.117	0.024	0.022	0.998	0.997	0.998	0.954	0.978

#### 4.2.2 Convergent and discriminant validity

Convergent Validity: We used average variance extracted (AVE) and composite reliability to assess it. The AVE values are: 0.794 (intrinsic motivation) and 0.8 (scientific writing self-efficacy), both higher than the recommended threshold of 0.50. Corresponding to the CR value, both 0.959 and 0.960 are acceptable convergence validity levels.

Discriminant Validity was investigated using the Fornell-Larcker test. According to Tables 5, 6, after standardisation with variance extraction, the correlation coefficients were 0.891 for intrinsic motivation and 0.894 for scientific writing self-efficacy, both of which exceeded that between these two variables (0.654, *p* < 0.01). These findings support adequate discriminant validity.

**Table 5:** Standardised factor loadings and convergent validity indices.

Item	Factor	Estimate	S.E.	C.R.	*p*	Standardised estimate	AVE	CR
Mot_1	Motivation	1				0.901	0.794	0.959
Mot_2	Motivation	1.036	0.054	19.126	<0.001	0.884		
Mot_3	Motivation	1.062	0.053	19.925	<0.001	0.898		
Mot_4	Motivation	1.042	0.054	19.373	<0.001	0.888		
Mot_5	Motivation	1.012	0.053	18.95	<0.001	0.88		
Mot_6	Motivation	0.985	0.05	19.763	<0.001	0.895		
Eff_1	Efficacy	1				0.879	0.800	0.96
Eff_2	Efficacy	1.065	0.058	18.402	<0.001	0.89		
Eff_3	Efficacy	1.048	0.056	18.74	<0.001	0.897		
Eff_4	Efficacy	1.065	0.057	18.761	<0.001	0.898		
Eff_5	Efficacy	1.079	0.057	18.82	<0.001	0.899		
Eff_6	Efficacy	1.044	0.055	19.066	<0.001	0.904		

**Table 6:** Discriminant validity based on the Fornell–Larcker criterion.

Construct	Intrinsic motivation	Self-efficacy
Intrinsic motivation	0.891	
Self-efficacy	0.654**	0.894

Diagonal elements correspond to square roots of AVE. Off-diagonal values are the inter-construct correlation coefficients. *p < 0.01.

Overall, the CFA results provided evidence to support the reliability, convergent validity and discriminant validity of the measurement model.

### 4.3 Assumptions testing

Before conducting the main analysis, normality tests and variances homogeneity test need to be carried out on the original data according to plan to ensure that the assumptions were satisfied.

#### 4.3.1 Normality

Normality of the samples at three levels: Intrinsic motivation, scientific writing self-efficacy; and within each group support conditions. According to Table 7, there was no significant deviation of non-normal distribution for the traditional support conditions. On the other hand, deep learning assisted condition exhibited serious non-normality of intrinsic motivation and self-efficacy.

**Table 7:** Normality test results for intrinsic motivation and scientific writing self-efficacy.

Writing support type	Dependent variable	Shapiro–Wilk W	df	*p*	Kolmogorov–Smirnov D	df	*p*
Traditional support	Intrinsic Motivation	0.983	100	0.222	0.077	100	0.151
DeepSeek-assisted	Intrinsic Motivation	0.966	100	0.011	0.110	100	0.004
Traditional support	Self - Efficacy	0.983	100	0.225	0.063	100	0.200
DeepSeek-assisted	Self - Efficacy	0.971	100	0.027	0.102	100	0.012

Although there was a balance in the design, equal cells and generally reliable results based on ANOVA-MANOVA tests when mild departures from normal distribution occurred at these levels. Therefore, we selected parametric analysis procedures here.

#### 4.3.2 Homogeneity of variance

Homogeneity of variance was examined using Levene’s test across the four writing-support-by-achievement cells. According to Table 8, there was no violation of the assumptions of homogeneity in variance for both scientific writing self-efficacy [*F*(3, 196) = 1.28; *p* = 0.283] and intrinsic motivation [F(3, 196) = 0.85; *p* = 0.467].

**Table 8:** Levene’s test of homogeneity of variance.

Dependent variable	F	df1	df2	*p*
Self-efficacy	1.279	3	196	0.283
Intrinsic motivation	0.852	3	196	0.467

### 4.4 Main and interaction effects of writing support type and academic achievement level

#### 4.4.1 MANOVA results

Multivariate Analysis of Variance (MANOVA) was used to assess whether there were differences in intrinsic motivation and scientific Writing Self-Efficacy among groups with different writing Support Types (DeepSeek-assisted, Traditional support), or according to Students’ grades.

An analysis showed that there was a substantial multivariate main effect of the write-support condition; Wilks’ *Λ* = 0.790, *p* < 0.001; therefore, these two groups were markedly different across all dependent measures. There is a notable multivariate main effect of the academic achievement level: Wilks’ *λ* = 0.830; *p* < 0.001; high and low-academic-achievement students differ significantly across all posttask psychological indicators (Wilks’ lambda). In addition, there was an impact on the relationship between writing assistance type and academic achievement; *p* < 0.001: The degree to which this condition differed significantly at various levels varied.

#### 4.4.2 Follow-up univariate ANOVAs

Follow-up univariate analysis of variance (ANOVA) was performed on the dependent variables separately.

In terms of intrinsic motivation, the main effect of writing support type is significant at *p* < 0.001 [*F*(1,198) = 259.3], while the main effect of academic achievement level also shows significance [*p* < 0.001; F(1,198) = 28.56]. There are no interaction effects.

Regarding the self-efficacy for scientific writing, both effects were significantly associated with writing assistance. Writing help was an independent predictor: *F*(1, 198) = 282.61, *p* < 0.001. The impact of student’s grade also contributed to it: F(1, 198) = 37.92, *p* < 0.001.

The results showed that, among the two types of writing support, post-task intrinsic motivation and scientific writing self-efficacy were affected differently by students’ level of learning. As shown in Table 9.

**Table 9:** The univariate ANOVA test of intrinsic motivation and scientific writing self-efficacy.

Dependent variable	Effect	F	*p*
Intrinsic Motivation	Writing support type	259.30	<0.001
Intrinsic Motivation	Academic achievement level	28.56	<0.001
Self - Efficacy	Writing support type	282.61	<0.001
Self - Efficacy	Academic achievement level	37.92	<0.001

#### 4.4.3 Interaction effect

The interaction effect of the Writing Support Type on dependent variable performance was strong in both cases. There is a significant interactive effect of scientific writing self-efficacy; F(1, 198) = 13.46, *p* < 0.001. The interaction effect of extrinsic motivation is also significant; F(1, 198) = 35.70, *p* < 0.001.

**Figure 1:**
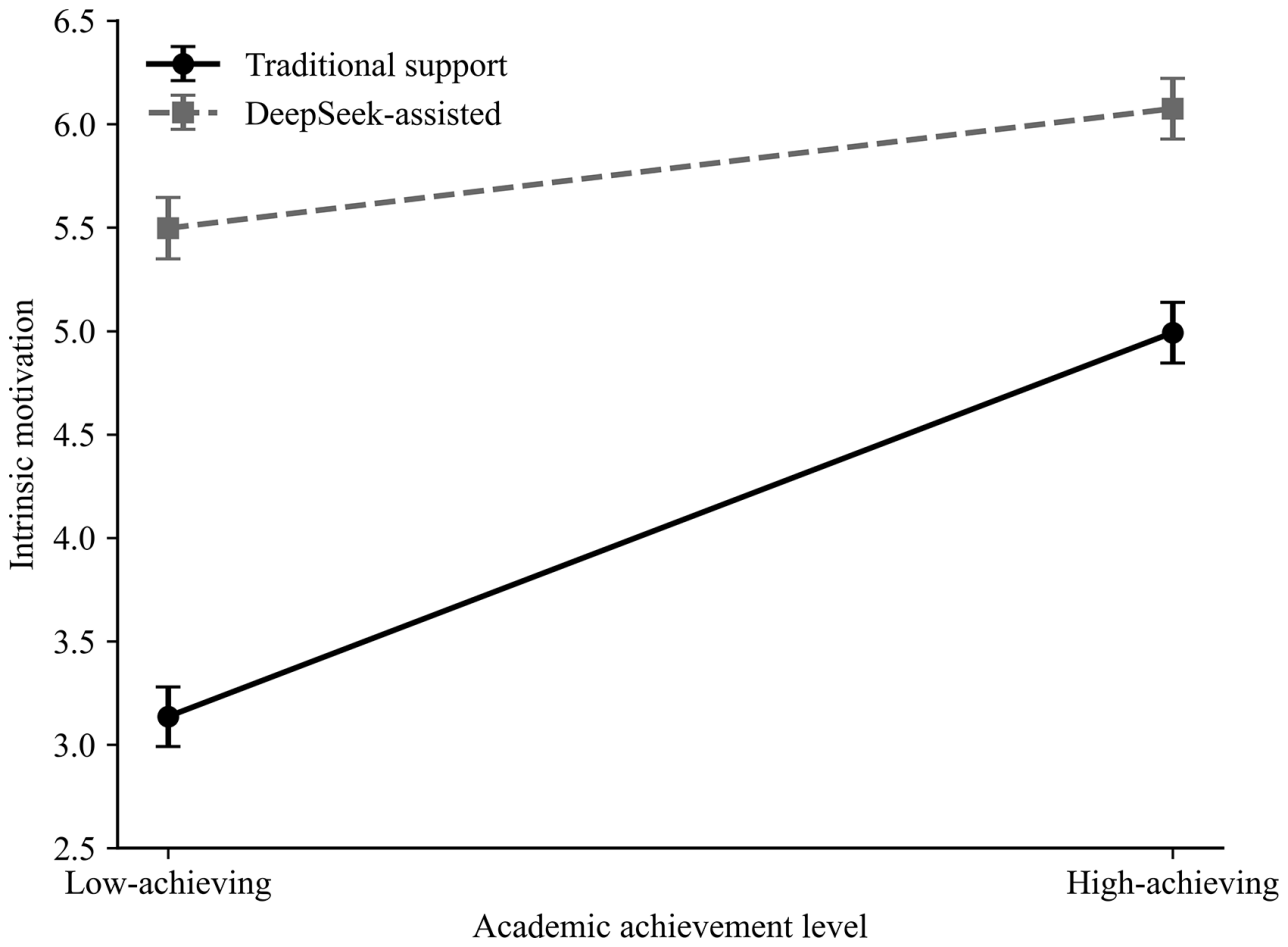
Interaction effect of writing support type and academic achievement level on intrinsic motivation.

**Figure 2:**
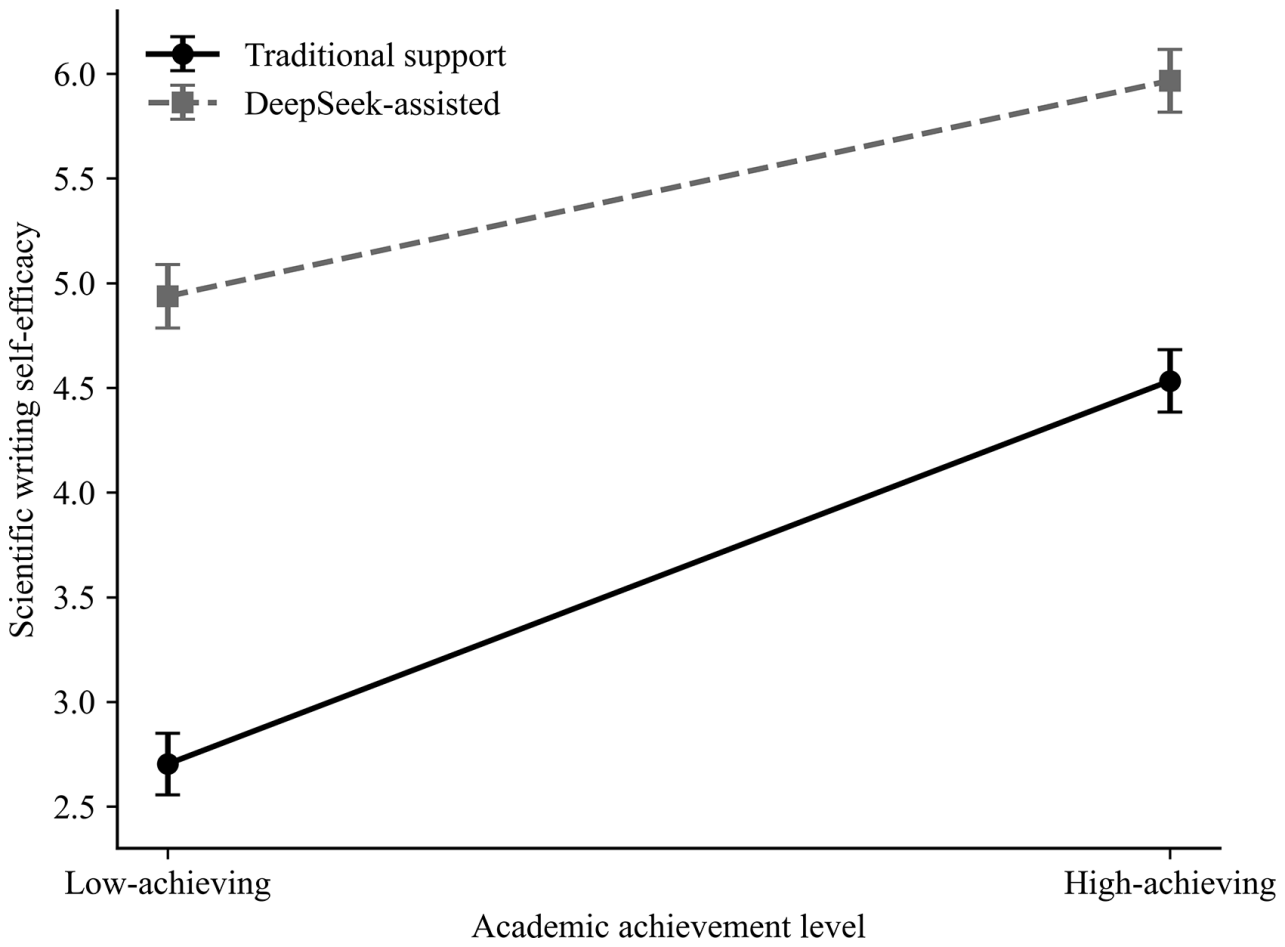
Interaction effect of writing support type and academic achievement level on scientific writing self-efficacy.

Based on Figures 1, 2, the student group assisted by DeepSeek had significantly higher intrinsic motivation and self-efficacy compared to the traditional support group at both learning outcomes. The interaction pattern further suggested that these support-condition differences were larger among low-achieving students than among high-achieving students.

Overall, there were clear main effects and interactions between Writing Support Type (WST) and Academic Achievement Level on both post-task Psychological Outcomes.

### 4.5 Control-adjusted results

To assess whether the observed group differences persisted following control for these confounding variables, analysis of covariance (ANCOVA) was used to explore scientific writing self-efficacy and intrinsic motivation, GPa, Task Familiarity, Grade Level, Gender, etc., were adjusted in the model.

#### 4.5.1 Scientific writing self-efficacy

Scientific writing self-efficacy: The ANCOVA showed that there was a significant main effect of writing assistance type. There is a difference between the two groups before and after adjustment for other factors; students in the DeepSeek-assisted group have significantly higher levels of scientific-writing self-efficacy compared to those under traditional support. There is a clear main effect of academic performance at the level *F*(1, 192) = 37.92, *p* < 0.001. The partial eta-square coefficient is 0.165: High-level students’ self-efficacy generally exceeded that of lower-level ones.

In addition, the interplay of writing support type and academic achievement level was notable, *F*(1, 192) = 13.46, *p* < 0.001, partial η^2^ = 0.066 indicated that there were differences among the support conditions based on students’ grades. GPA exhibited a slight but statistically significant impact in terms of covariance, F(1, 192) = 4.71, *p* = 0.031, partial η^2^ = 0.024. However, task familiarity, grade level and gender were not statistically significant.

Levene’s test for equality of error variances did not show significance, *F* (3,196) = 1.28, *p* = 0.283, so it could be assumed to meet the homogeneity assumption in this model.

Based on the estimated marginal means, it can be observed that the students who received traditional support and achieved at a lower level had the lowest scientific writing self-efficacy score (mean of 2.703), while those provided with DeepSeek assistance but achieved more highly scored high-scoring groups (deepseekassisted-high-achievement) significantly higher scores (M = 5.967). The other two experimental groups are slightly below average, which suggests they receive both forms of support or have varying levels of achievement. This type is consistent with its strong interaction effect.

The interaction pattern indicated that the advantage of DeepSeek-assisted support over traditional support was more pronounced among low-achieving students than among high-achieving students (see Tables 10, 11).

**Table 10:** ANCOVA results for scientific writing self-efficacy controlling for GPA, task familiarity, grade level, and gender.

Effect	F	*p*	Partial η^2^
Writing support type	282.61	<0.001	0.595
Academic achievement level	37.92	<0.001	0.165
Writing support type × Academic achievement level	13.46	<0.001	0.066
GPA	4.71	0.031	0.024
Task familiarity	0.79	0.377	0.004
Grade level	0.14	0.710	0.001
Gender	0.39	0.533	0.002

**Table 11:** Estimated marginal means of scientific writing self-efficacy by writing support type and academic achievement level.

Writing support type	Academic achievement level	M	SE	95% CI
Traditional support	Low	2.703	0.147	[2.412, 2.993]
Traditional support	High	4.533	0.149	[4.240, 4.827]
DeepSeek - assisted	Low	4.937	0.152	[4.637, 5.238]
DeepSeek - assisted	High	5.967	0.150	[5.672, 6.262]

Estimated marginal means were computed after controlling for GPA, task familiarity, grade level, and gender.

#### 4.5.2 Intrinsic motivation

With respect to intrinsic motivation, the ANOVA also found that there was a significant main effect of writing assistance type; *F*(1, 192) = 259.30, *p* < 0.001, η^2^p = 0.575. In particular, Students’ scores were lower in the deep assistance group and closer to those in the control group after adjusting for covariates. There was an evident interaction effect between academic ability level and intrapersonal motivation: *F*(3, 576) = 4.08, *p* = 0.000. There was no obvious interactive effect on extrinsic motivation (*p* > 0.05).

The relationship between type of writing support and academic performance was also substantial; F(1, 192) equals 35.70, *p* < 0.001, η^2^p = 0.157 at high levels of ability, showing that there is a variation in this connection with students’ abilities. None of the covariates reached statistical significance, including GPA, F(1, 192) = 0.52, *p* = 0.472; task familiarity, F(1, 192) = 0.01, *p* = 0.906; grade level, F(1, 192) = 0.14, *p* = 0.706; and gender, F(1, 192) = 1.80, *p* = 0.181.

Levene’s test was not significant; *F*(3, 196) = 0.85, *p* = 0.467, which indicated that there met the homogeneous assumption of this model.

The estimate of marginal means indicated that intrinsic motivation was the lowest among the traditional support group with low achievement level (M = 3.136, SE = 0.144), followed by the same conditions under high achievement level (M = 4.992, SE = 0.146); deep-seek-assisted groups also experienced a relatively lower amount, particularly at both low-achievement levels: (M = 5.497; SE = 0.149) [5.203 ~ 5.792]; Also note at higher achievements: (M = 6.075; SE = 0.147) [5.785 ~ 6.365]. The same kind of situation exists in this way, too.

**Figure 3:**
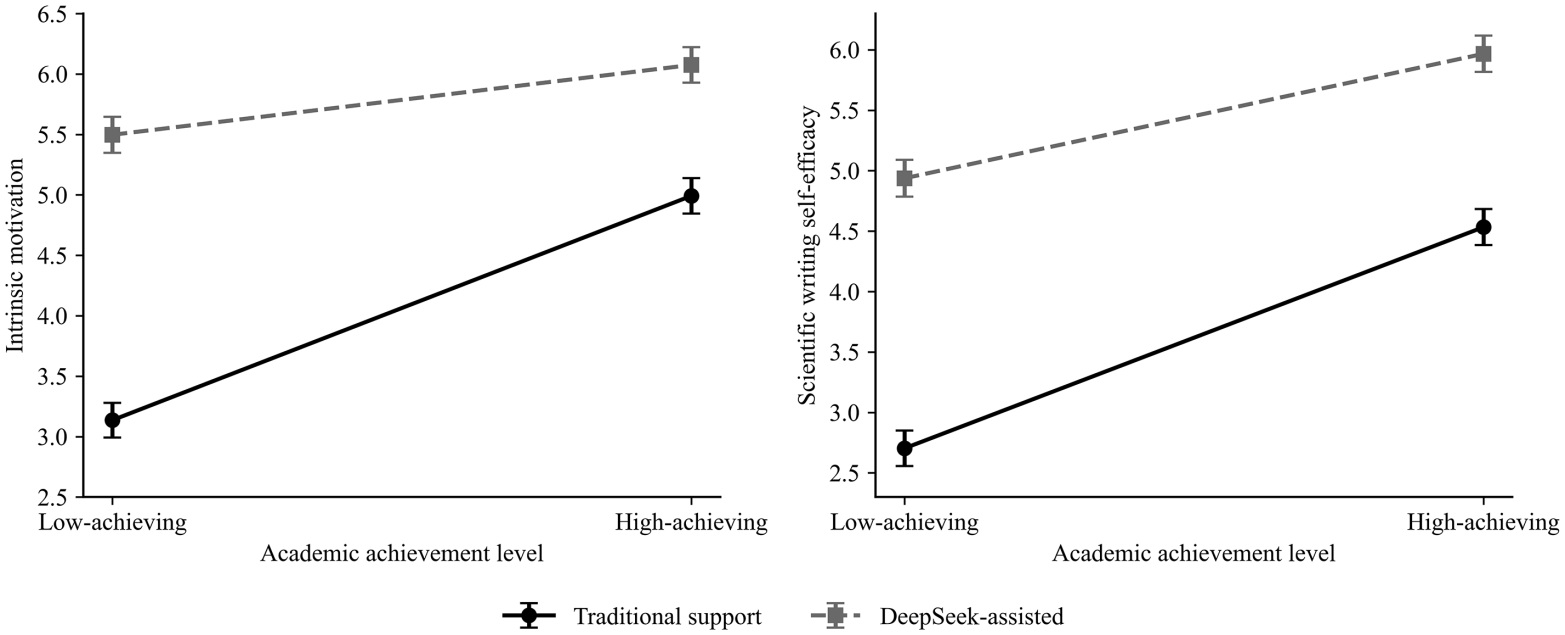
Covariate-adjusted post-task intrinsic motivation, scientific writing self-efficacy, writing support type (WST), and academic achievement level (AAL).

Similarly, the positive effect of DeepSeek-assisted support on intrinsic motivation was stronger for low-achieving students, suggesting that AI-assisted support may be particularly beneficial for students with lower prior academic achievement (see Figure 3; Tables 12, 13).

**Table 12:** ANCOVA results of intrinsic motivation adjusted by GPA, task familiarity, grade level and gender.

Effect	F	*p*	Partial η^2^
Writing support type	259.30	<0.001	0.575
Academic achievement level	28.56	<0.001	0.129
Writing support type × Academic achievement level	35.70	<0.001	0.157
GPA	0.52	0.472	0.003
Task familiarity	0.01	0.906	0.000
Grade level	0.14	0.706	0.001
Gender	1.80	0.181	0.009

**Table 13:** Estimated marginal means of intrinsic motivation by writing support type and academic achievement level.

Writing support type	Academic achievement level	M	SE	95% CI
Traditional support	Low	3.136	0.144	[2.851, 3.420]
Traditional support	High	4.992	0.146	[4.704, 5.280]
DeepSeek-assisted	Low	5.497	0.149	[5.203, 5.792]
DeepSeek-assisted	High	6.075	0.147	[5.785, 6.365]

Estimate of the marginal mean after controlling for GPA, task familiarity, grade level and gender.

### 4.6 Mediation analysis

To further examine whether intrinsic motivation mediated the relationship between writing support type and scientific writing self-efficacy, a simple mediation model (Model 4) was tested using PROCESS macro Version 4.2. Writing support type was specified as the independent variable, intrinsic motivation as the mediator, and scientific writing self-efficacy as the dependent variable. Indirect effects were estimated using 5,000 bootstrap samples.

The Table 14 showed that writing support type significantly predicted intrinsic motivation, *b* = 1.717, *SE* = 0.137, *t* = 12.52, *p* < 0.001, 95% CI [1.446, 1.987], indicating that students in the DeepSeek-assisted condition reported higher intrinsic motivation than those in the traditional-support condition. In addition, intrinsic motivation significantly predicted scientific writing self-efficacy when writing support type was controlled, *b* = 0.338, *SE* = 0.064, *t* = 5.24, *p* < 0.001, 95% CI [0.211, 0.465].

**Table 14:** Mediation analysis results.

Path	*b*	*SE*	*t*	*p*	95% CI
writing support type → intrinsic motivation	1.717	0.137	12.52	<0.001	[1.446, 1.987]
intrinsic motivation → scientific writing self-efficacy	0.338	0.064	5.24	<0.001	[0.211, 0.465]
writing support type → scientific writing self-efficacy(direct)	1.271	0.166	7.64	<0.001	[0.943, 1.599]
Indirect effect via intrinsic motivation	0.579	0.116	—	—	[0.373, 0.825]

After intrinsic motivation was entered into the model, the direct effect of writing support type on scientific writing self-efficacy remained significant, *b* = 1.271, SE = 0.166, *t* = 7.64, *p* < 0.001, 95% CI [0.943, 1.599], indicating partial mediation. Moreover, the indirect effect of writing support type on scientific writing self-efficacy through intrinsic motivation was significant, *b* = 0.579, BootSE = 0.116, 95% CI [0.373, 0.825], because the bootstrap confidence interval did not include zero.

Overall, the findings support a mediation pathway from writing support type to scientific writing self-efficacy through intrinsic motivation.

**Figure 4:**
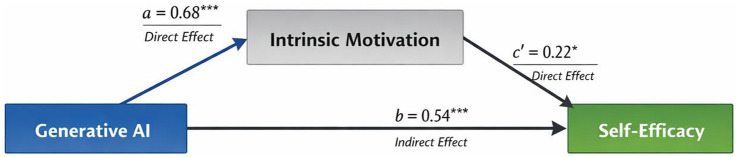
Mediation model linking writing support type, intrinsic motivation, and scientific writing self-efficacy.

As illustrated in Figure 4, writing support type was associated with both self-efficacy directly and through intrinsic motivation. It met the criteria for a partial mediation model.

## 5 Discussion

The present study examined whether DeepSeek-assisted scientific writing was associated with university students’ intrinsic motivation and scientific writing self-efficacy, and whether these associations varied by academic achievement level.

### 5.1 Deepseek assisted writing showed an increase in intrinsic motivation and self-efficacy

The first major result was that, compared to the non-deep-seeker group, students in the DeepSeek-assisted condition showed higher levels of intrinsic motivation and scientific writing self-efficacy. This pattern can be seen from the description, MANOVA and ANOVA results; also from the covariate-control analysis results. Even when adjusting for GPA, task familiarity, grade level, and gender, the DeepSeek-assisted condition still had a relatively high post-task score in both aspects. Based on this, it can be concluded that the group differences did not diminish after controlling for all relevant factors.

This result is in broad agreement with both SDT and the self-efficacy theory. In the context of SDT, generator-assistive AI could boost learners’ internal interest via more interactive experiences and personalisation features. Students will be more driven by the internalisation of autonomy and competence when they get quick answers, personalised hints, etc., to satisfy their growing need for intrinsic motivation. AI does not offer true human-relatedness at all levels of SDT; however, its responsive and non-pressure-inducing behaviour might generate an artificial feeling of support that motivates continuous participation.

Based on self-efficacy Theory, AI-assisted Writing might correspond to a higher belief in one’s ability because it offers real-time help, reduces task uncertainties, helps build an increasing sense of achievement. This support can help boost students’ belief in their ability to comprehend the task, organise ideas clearly, and write a good academic work. Therefore, in terms of this type of generation technology, some writers believe that they will assist content creators more significantly than enhance their capabilities as authors.

Generative Artificial Intelligence’s education-related significance extends to more factors than simply enhancing production speed and improving results, according to these observations. Currently most discussions centre around productivity, personalisation and achievement. The present results suggest that AI tools may also be associated with students’ motivational beliefs and self-perceptions. The all-round function it assumes, especially at the university level when persistence and motivation tend to correlate with a student’s sense of capability and interest in investing energy in study.

### 5.2 High-achieving students reported higher overall motivation and self-efficacy

A substantial primary Effect in academic performance levels was revealed to a considerable extent. Across all conditions, high-achieving students reported greater intrinsic motivation and scientific writing self-efficacy than low-achieving students. Based on theory, it conforms to the existing research findings that show a correlation between previous educational attainment and students’ sense of capability belief, optimistic attitude towards learning outcomes, etc.

High-achieving students have had richer experiential learning of success and failure; they are familiar with better ways to learn academically; they believe that they possess greater capabilities for self-efficacy. Such advantages can further strengthen one’s sense of self-efficacy and interest in learning among many demands. In terms of performance, the lower-performing students have more difficulty understanding them or are unwilling to participate fully. Therefore, it is possible that there are overall differences in students’ learning backgrounds and self-efficacy among various groups based on this observation.

At the same time, there is also a need to provide interpretive basis for understanding the support-condition difference at this stage. Despite having a favourable overall psychological profile, the interaction analysis revealed that there were no greater support-condition differences among highly performing students due to AI assistance. Instead, there were greater disparities between high-and low-scoring students. This matter serves as a reference for this research.

### 5.3 Stronger associations among low-achieving students suggest the equalising potential of generative AI

Among the key results were a substantial effect of the combination of different types of writing assistance with student interest and scientific writing proficiency at various levels. Simple-effect analysis shows that, relative to the traditional support conditions, students in either group received assistance via DeepSeek reported significantly better learning effects. However, the effect was much more pronounced for lower achieving students. The above situation appeared in both ANOVA, ANCOVA, and moderation analysis.

This indicates that, to some extent, Generative AI has equalising capabilities during the learning process. Low-achieving students usually lack both internal and external conditions to conduct independent learning autonomously. They may have greater difficulties comprehending; they ask for help more often when having trouble understanding and need less targeted assistance individually at present. Generative AI can partially offset some of the shortcomings through real-time explanation, clarify confusion, generate instances, provide examples, and offer on-demand, nonjudgmental assistance. Such characteristics can make them more suitable as support for some students with greater difficulty but less inclined towards seeking assistance through regular class settings.

This pattern can also be understood from both the perspective of scaffolding and Cognitive Load Theory. The students whose understanding is less solid can get more special care and support, thus reducing their sense of helplessness caused by incomplete understanding. AI-assisted writing can assist students in organising materials, polishing expressions, focusing their minds, reducing some requirements of the complicated structure of scientific essays by providing support. As these barriers are removed, lower-achieving students may have more positive engagement in productive ways on the tasks; as a result, they will feel motivated and confident.

However, one should be cautious about interpreting the “equally promoting development” claim cautiously. At present, generative artificial intelligence does not eliminate educational inequalities and has also not achieved sustained reduction in academic disparities over time. Actually, it shows that in these circumstances, compared to high-performing students, DeepSeek assisted learning is more likely to provide greater psychological benefits for low-performers after completing a task. It still has some utilitarian values at present. Given that well-coordinated AI assistance can be beneficial to students with weaker foundations. Therefore, the education of this technology may be particularly beneficial for lower-achieving students.

### 5.4 Intrinsic motivation as a partial mediating pathway

Mediation analysis revealed that Intrinsic Motivation partly mediate the relationship between writing support type and scientific writing self-efficacy. The above results provide a detailed exploration of the connection between students’ psychological states and using AI-assisted writing support.

One possibility for interpretation is that, initially, generative AI constructs students’ experiences of the learning process. When students perceive that a task has interest, non-demand or profit in relation, they are likely to acquire more intrinsic motivations. Such an motivation can stimulate people’s high interest, determination and proactive attitude to solve some issues - therefore possibly increasing his sense of competence over time. That is, as a result of the help provided by artificial intelligence in the learning process, students’ sense of capability may increase to some extent.

In terms of fact, it was a part-of-the-mediation process according to theory as well. It implies that the pathway of intrinsic motivation may not be sufficient on its own. Generative AI may also be associated with self-efficacy through other mechanisms, such as immediate feedback, reduced ambiguity, greater perceived task clarity, lower perceived cognitive load, or a stronger sense of support during task completion. Therefore, future research needs to explore more mediators beyond perceived competence, cognitive engagement, learning satisfaction, help-seeking confidence, and decreased academic anxiety.

At this time, the interpretation of the mediated outcome should be made cautiously. Due to all focal variables being assessed after task completion without including multiple-measurement designs at different times, it cannot confirm an actual causative mechanism but merely demonstrates that such a relationship exists. Therefore, the results can be more reliably explained to support a reasonable motivational path and then tested in longitudinal or pretest-postest designs.

### 5.5 Covariate-controlled analyses and the robustness of the findings

The other advantage of this study is that most patterns remain significantly strong even when controlled by GPA, task familiarisation, teaching phase, gender or grades. Therefore, the supported condition differences may not have been entirely due to variations in the measured background variables incorporated into these models. GPA has shown some significance; otherwise none of the others are statistically significant in this self-efficacy model. None of the covariates were significant in the intrinsic motivation model. Central main effect and interaction effects were still consistent with those of the unadjusted data.

These results strengthen belief that the observed phenomenon is not an artefact of the measured background properties. However, they should not be taken to mean that all relevant confounding has been ruled out. Other unknown factors - including initial motivation at the start of this work; Prewriting apprehension and previous exposure to AI tools may also influence these results. Therefore, although covariate-controlled analyses increase the reliability of the results presented here; however, none are free from threats to their credibility.

### 5.6 Theoretical contributions

This paper has made some theoretical contributions.

First of all, extend the study on generative AI in education to focus more closely on psychological effects rather than just results. The results show how integrating generation-AIs may affect students’ sense of motivation and interest in studying indirectly through an educational lens on effective teaching.

Secondly, this article combines Self-determination Theory, self-efficacy theory, and a scaffolding/Cognitive Load Theory approach to discuss how AI-supported Learning affects students. A higher level of intrinsic motivation has been reported by subjects assisted deep-seeker, which aligns with the notion that an autonomous-learning environment helps develop intrinsic motivation. The higher scientific writing self-efficacy mentioned under the same condition is consistent with this perspective: Timely support, task guidance and successful experience make learning more efficient for students. A relatively obvious trend was also observed in the high-performing group; namely, Scaffolding aid might be most effective for those struggling more significantly with tasks at hand.

Third, the modulated results provide us with a richer Understanding of who might benefit more from Generative Artificial Intelligence. Rather than making all associations between learners uniformly, this study proposes that different aspects of the learners’ characteristic affect the degree of relationship among artificial intelligence-based assistance and psychological effects. A learner-centred viewpoint will be necessary for future theories of AI-assisted education’s development.

### 5.7 Practical implications

The results also have several practical applications.

First, teachers and educational institutions could view the integration of generation-oriented AI teaching assistent (TaaS) as structural learning aids rather than merely supplementary or auxiliary technology. Guided in time, these items may attract students’ attention to learn more actively about how difficult they will be with certain contents.

The more obvious pattern found in lower-performing students indicates that it could be most effective for remedial, transitional, or support-based education. Reiterate the answers given by some who hesitate to speak; for others anxious for instant response, provide actual updates through the network environment. Therefore, the concept of an all-inclusive support structure for individuals that can scale according to available human resources exists in some situations.

Thirdly, Implementation is required to accompany pedagogical support. Generative AI’s benefits will not appear on their own. Students require assistance in using the intelligent product reasonably to question its output independently and make learning more meaningful through integrated applications of artificial intelligence technology. Therefore, AI literacy, prompt literacy and mindful instructional Design are still necessary to achieve optimal results without over-reliance or surface-level application.

Its main application is also about improving students’ psychology, not to show an improvement in their writing ability objectively. Therefore, institutions need to be wary of presuming a positive correlation between motivation and self-efficacy and better-written works without collecting any data;

### 5.8 Limitations and directions for future research

Some problems exist as follows.

First, this study did not incorporate pretests of intrinsic motivation or scientific writing self-efficacy. Therefore, no evidence of difference in the underlying psychological traits at baseline was obtained; hence, within-person changes during training cannot be proven through this method. The findings should therefore be interpreted as post-task differences between groups, not as directly observed increases from pretest to posttest. Future research can design a pre-test-post test comparison using an AI-assisted Learning intervention.

Second, the study used a quasi-experimental design conducted in an authentic course context rather than a fully randomized blinded experiment. Participants knew if they were using DeepSeek or traditional sources; thus, blindness was impossible due to the characteristics of this kind of writing assistance. This recognition may have brought out-expectancy effect or demand characteristics. In the future, other Design Strategies to Reduce Expectancy Bias, Such As Alternative Comparison Conditions, Delayed Exposure Designs Or Repeated Use Designs.

Thirdly, there may be a phenomenon of novelty. Among the more favourable responses under DeepSeek assistance, some might be driven by curiosity and interest among students who use new technologies, which is not necessarily related to the inherent benefits of AI-supported learning. To investigate if the psychological benefits remain after there has been a reduction in the novel-effect based on our present research results.

Fourth, the current research used self-report measures to assess intrinsic motivation and self-efficacy. Although these are effective educational models; as self-reports, they can be affected by factors such as social conformity and subjectively interpreting the tasks. In the future, we need to combine self-reports with behavioural data and processes to obtain more complete information about AI-assisted learning from observation methods.

Fifth, this study did not cover an objective measure of writing quality or academic outcomes. Therefore, there are no data to suggest that generative AI helped improve the quality of students’ written work or their overall academic performance. Future research may include rubric-based writing evaluation methods; Blind Ratings for Texts; Revision-Quality Indicators or Course-Based Performance Outcomes to see if these changes lead to measurable benefits in performance improvement.

The Sixth is that the study has selected an individual group based on certain samples, environments, etc., which reduces its generality. The consequences that generative AI will have differ among various fields, ages, schools, countries, cultures, and tasks. Therefore, in the future research, it may vary this pattern according to diverse educational contexts.

The seventh is that despite mediation identification of intrinsically motivated students, other pathways need to be explored in-depth. Future research will further investigate whether perceived competence, academic enjoyment, cognitive involvement, help-seeking behaviour, task clarification, or reduced anxiety contribute to the relationships among artificial intelligence-assisted writing and students’ psychological effects.

At last, although it shows the equalising power of generative AI, further research is needed to investigate its boundary condition at present. AI may help less capable learners more appropriately in some instruction arrangements than it can. Factors like digital access, prompt quality, teacher guidance, confidence in AI, and critical skills in using AI will determine to what extent and whether these effects occur positively or negatively.

### 5.9 Overall interpretation

Overall, the results suggest that DeepSeek-assisted writing is positively correlated with better post-Task psychological effects in college students, specifically higher levels of intrinsic motivation and improved scientific writing Self-efficacy. Association was observed in these two groups of students; however, it was greater among the lower-scoring learners. Moreover, intrinsic motivation was identified as a possible partial path from the Writing Support Type to Self-Efficacy.

The above data show that in addition to serving the purpose of generating content, Generative AI could act as an ally and friend during education when required by the environment. However, at this time, its conclusions must also be within the design range; that is to say, these results are merely self-reported post-training psychological effects under specific circumstances and cannot be regarded as final cause-and-effect relationships or true performance enhancements. Although there are some limitations, the research does suggest that generative AI has considerable potential as an auxiliary tool in undergraduate education; it is most suitable for those with greater motivation and confidence issues due to demanding courses.

## 6 Conclusion

This study examined whether DeepSeek-assisted scientific writing was associated with university students’ intrinsic motivation and scientific writing self-efficacy, and whether these associations differed by academic achievement level. The research results indicated that the deepseek-assisted group had significantly stronger intrinsic motivation and self-efficacy after learning compared to the general support treatment. The support-condition differences were found to be statistically significant in subsequent control of GPa, Task Familiarity, Grade Level, Gender and other factors. In terms of their background characteristics, these conditions are consistent.

There is also a moderate effect of the correlation between students’ academic performance and changes in motivation to write. High-achieving students, although generally reporting greater motivation and self-efficacy in all two conditions, showed particularly significant support-condition effects for AI-supported writing among low-achievers. Based on this pattern, generative AI can serve as an aid for students requiring additional assistance in challenging their studies; there are also considerations of equality-enhancing opportunities it brings through reduced motivational and self-esteem gaps between learners.

In addition, intrinsic motivation partly mediated the relationship among writing support type, self-efficacy of scientific writing. The above result provides an accessible mechanism linking AI-assisted writing to higher confidence beliefs: Students, who find these tasks interesting and satisfying in some way, may also believe they are better equipped for them. At the same time, it needs to be interpreted with caution that there is no strong causal claim on changes in psychology through this mediated relationship at present.

In general terms, there is a positive correlation between the use of generative AI-assisted writing support and students’ post-task psychological state, such as higher levels of intrinsic motivation and improved science-writing self-efficacy. The results of particular relevance to low-achieving students have larger differences due to their support conditions. With appropriate instructional Design and Critical pedagogy guidance, generative AI can be considered as a useful Supplemental material to help create more favourable conditions of support, motivation, and possibly inclusivity for students’ learning in higher education. These conclusions need to be understood within the scope of this study, which includes its quasi- experimental design, dependence on self-report measurement, lack of pretest data, and focus only on one particular teaching environment.

## Data Availability

The raw data supporting the conclusions of this article will be made available by the authors, without undue reservation.

## References

[ref1] Bandura A. (1997). Self-Efficacy: The Exercise of Control. New York, NY: W.H. Freeman and Company.

[ref2] Bouzar D. A. (2024). ChatGPT and academic writing self-efficacy: unveiling correlations and technological dependency among postgraduate students. Arab World Engl. J. 1, 225–236. doi: 10.24093/AWEJ/CHATGPT.15

[ref3] Chen S. Cheung A. C. K. (2025). Effect of generative artificial intelligence on university students’ learning outcomes: a systematic review and meta-analysis. Educ. Res. Rev. 49:100737. doi: 10.1016/j.edurev.2025.100737

[ref4] Chen J. Mohamed Mokmin N. A. Su H. J. (2025). Integrating generative artificial intelligence into design and art courses: effects on student achievement, motivation, and self-efficacy. Innov. Educ. Teach. Int. 62, 151–165. doi: 10.1080/14703297.2025.2503857

[ref5] Chen R. Wu Y. Chen Z. Zhou P. (2025). Advancing educational equity in rural China: the impact of AI devices on teaching quality and learning outcomes for sustainable development. Front. Psychol. 16:1588047. doi: 10.3389/fpsyg.2025.1588047, 41267767 PMC12626958

[ref6] Deci E. L. Ryan R. M. (2000). The "what" and "why" of goal pursuits: human needs and the self-determination of behavior. Psychol. Inq. 11, 227–268. doi: 10.1207/S15327965PLI1104_01

[ref7] Deci E. L. Ryan R. M. (2013). Intrinsic Motivation and Self-Determination in Human Behavior. New York, NY: Springer.

[ref8] Gabriel S. Generative AI and educational (in) equity. In International Conference on AI Research (2024) 133–142.

[ref9] Golombek C. Klingsieck K. B. Scharlau I. (2018). Assessing self-efficacy for self-regulation of academic writing. Eur. J. Psychol. Assess. 35, 751–761. doi: 10.1027/1015-5759/a000452

[ref10] Hao L. Yusoff S. M. Tian K. (2026). Impacts of AI-enhanced task-based learning on EFL postgraduates’ higher order thinking skills and English academic writing self-efficacy. J. Engl. Acad. Purp. 80:101652. doi: 10.1016/j.jeap.2026.101652

[ref11] Huang J. Mizumoto A. (2024). Examining the effect of generative AI on students’ motivation and writing self-efficacy. Digit. Appl. Linguist. 1:102324. doi: 10.29140/dal.v1.102324

[ref12] Hwang Y. Wu Y. (2025). The influence of generative artificial intelligence on creative cognition of design students: a chain mediation model of self-efficacy and anxiety. Front. Psychol. 15:1455015. doi: 10.3389/fpsyg.2024.1455015, 39931512 PMC11808137

[ref13] Jin Y. Yan L. Echeverria V. Gašević D. Martinez-Maldonado R. (2025). Generative AI in higher education: a global perspective of institutional adoption policies and guidelines. Comput. Educ. Artif. Intell. 8:100348. doi: 10.1016/j.caeai.2024.100348

[ref14] Khayr R. Karawani H. Banai K. (2023). Implicit learning and individual differences in speech recognition: an exploratory study. Front. Psychol. 14:1238823. doi: 10.3389/fpsyg.2023.1238823, 37744578 PMC10513179

[ref15] Kramer L. Lüdtke S. Freund P. A. (2025). Need for cognition, academic self-efficacy and parental education predict the intention to go to college—evidence from a multigroup study. Front. Psychol. 16:1487038. doi: 10.3389/fpsyg.2025.1487038, 39973946 PMC11835861

[ref16] Liu C. Zhu K. Zhang Z. Ding Y. (2025). The impact of Chinese undergraduates’ perceived acceptance of AI technology on academic achievement: the mediating role of critical thinking. Front. Psychol. 16:1727037. doi: 10.3389/fpsyg.2025.1727037, 41459280 PMC12741099

[ref17] Millin T. Millin M. Pearce J. (2020). Unpacking the efficacy of reading to learn using cognitive load theory. J. Acad. Lang. Learn. 14, 113–126. Available online at: https://journal.aall.org.au/index.php/jall/article/view/693

[ref18] Mohamed A. M. Shaaban T. S. Bakry S. H. Guillén-Gámez F. D. Strzelecki A. (2024). Empowering the faculty of education students: applying AI’S potential for motivating and enhancing learning. Innov. High. Educ. 50, 587–609. doi: 10.1007/s10755-024-09747-z

[ref19] Nawal A. F. (2018). Cognitive load theory in the context of second language academic writing. High. Educ. Pedagog. 3, 385–402. doi: 10.1080/23752696.2018.1513812

[ref20] Paas F. Renkl A. Sweller J. (2003). Cognitive load theory and instructional design: recent developments. Educ. Psychol. 38, 1–4. doi: 10.1207/s15326985ep3801_1

[ref21] Pang W. Wei Z. (2025). Shaping the future of higher education: a technology usage study on generative AI innovations. Information 16:95. doi: 10.3390/info16020095

[ref22] Pellas N. (2023). The effects of generative AI platforms on undergraduates’ narrative intelligence and writing self-efficacy. Educ. Sci. 13:1155. doi: 10.3390/educsci13111155

[ref23] Qian Y. (2025). Pedagogical applications of generative AI in higher education: a systematic review of the field. TechTrends 69, 1105–1120. doi: 10.1007/s11528-025-01100-1

[ref24] Ryan R. M. Deci E. L. (2000). Self-determination theory and the facilitation of intrinsic motivation, social development, and well-being. Am. Psychol. 55:68. doi: 10.1037/0003-066x.55.1.68, 11392867

[ref25] Sweller J. (1988). Cognitive load during problem solving: effects on learning. Cogn. Sci. 12, 257–285. doi: 10.1016/0364-0213(88)90023-7

[ref26] Tang S. (2025). The effectiveness of a generative AI-based literature module on motivation and self-efficacy among higher vocational students: a quasi-experimental study. Educ. Insights 2, 67–73. doi: 10.70088/3c2ayy74

[ref27] Tanveer I. Iqbal S. Hussain A. (2024). Examining the impact of AI based Chatbots on academic self-efficacy and self-regulation among university students. J. Dev. Soc. Sci. 5, 468–477. doi: 10.47205/jdss.2024(5-II-S)45

[ref28] Wilmers N. (2024). Generative AI and the future of inequality. [preprint. doi: 10.21428/e4baedd9.777b7123

[ref29] Xia Q. Li W. Yang Y. Weng X. (2025). A systematic review and meta-analysis of the effectiveness of generative AI on students' motivation and engagement. Comput. Appl. Artif. Intell. 38:100455. doi: 10.1016/j.caeai.2025.100455

[ref30] Xie Y. Wang S. (2025). Generative artificial intelligence in entrepreneurship education enhances entrepreneurial intention through self-efficacy and university support. Sci. Rep. 15:24079. doi: 10.1038/s41598-025-09545-3, 40618006 PMC12228775

[ref31] Xu Q. Liu Y. Li X. (2025). Unlocking student potential: how AI-driven personalized feedback shapes goal achievement, self-efficacy, and learning engagement through a self-determination lens. Learn. Motiv. 91:102138. doi: 10.1016/j.lmot.2025.102138

[ref32] Yilmaz R. Karaoglan Yilmaz F. G. (2023). The effect of generative artificial intelligence (AI)-based tool use on students’ computational thinking skills, programming self-efficacy and motivation. Comput. Educ. Artif. Intell. 4:100147. doi: 10.1016/j.caeai.2023.100147

[ref33] Zhang L. Xu J. (2025). The paradox of self-efficacy and technological dependence: unraveling generative AI'S impact on university students' task completion. Internet High. Educ. 40:100978. doi: 10.1016/j.iheduc.2024.100978

[ref34] Zimmerman B. J. Bandura A. (1994). Impact of self-regulatory influences on writing course attainment. Am. Educ. Res. J. 31, 845–862. doi: 10.3102/00028312031004845

